# Association between healthy lifestyle combinations and periodontitis in NHANES

**DOI:** 10.1186/s12903-024-03937-z

**Published:** 2024-02-04

**Authors:** J.N. Xu, Y.Q. Huang, J. Wang, H.L. Wang, C. Sun, Wentao Shi, Xinquan Jiang

**Affiliations:** 1 Department of Prosthodontics, Shanghai Ninth People’s Hospital, Shanghai Jiao Tong University School of Medicine, Shanghai, China; 2 College of Stomatology, Shanghai Jiao Tong University, No. 639 Zhizaoju Road, Huangpu District, Shanghai, China; 3 National Center for Stomatology, Shanghai, China; 4 National Clinical Research Center for Oral Diseases, Shanghai, China; 5 Shanghai Key Laboratory of Stomatology, Shanghai, China; 6 Shanghai Research Institute of Stomatology, Shanghai, China; 7 School of public health, Shanghai Jiao Tong University School of Medicine, Shanghai, China; 8 Clinical Research Unit, Shanghai Ninth People’s Hospital, Shanghai Jiao Tong University School of Medicine, No. 639 Zhizaoju Road, Huangpu District, Shanghai, 200011 China

**Keywords:** Healthy lifestyle, Cross-sectional studies, Periodontitis, NHANES, Combined effect

## Abstract

**Background:**

Periodontitis is closely associated with chronic systemic diseases. Healthy lifestyle interventions have health-enhancing effects on chronic systemic disorders and periodontitis, but the extent to which healthy lifestyle combinations are associated with periodontitis is unclear. Therefore, this study aimed to investigate the association between periodontitis and different healthy lifestyle combinations.

**Methods:**

5611 participants were included from the National Health and Nutrition Examination Survey (NHANES, 2009–2014). Six healthy lifestyles factors were defined as fulfilling either: non-smoking, moderate drinking, moderate body mass index (BMI), physical activity, healthy sleep and appropriate total energy intake. Then, the adjusted logistic regression models were performed to identify the association between the periodontitis and the scoring system composed of six lifestyles (0–6 scale). Finally, different scenarios were dynamically and randomly combined to identify the optimal and personalized combination mode.

**Results:**

Higher healthy lifestyle scores were significantly associated with lower periodontitis prevalence (*p* < 0.05). Four lifestyle factors (smoking, drinking, BMI, and sleep) significantly varied between the periodontitis and healthy groups (*p* < 0.05). Smoking was considered as a strong independent risk factor for periodontitis in both former and current smokers. Results further indicated that the combination of these four lifestyles played the most essential role in determining the magnitude of periodontitis occurrence (odds ratio [OR]: 0.33; 95% confidence interval [CI]: 0.21 to 0.50). In the total population, the majority of three lifestyle combinations outperformed the two combination models, whereas the two-combination of nonsmoking-drinking (OR: 0.39; 95% CI: 0.27 to 0.58) had relatively lower periodontitis prevalence than the three-combination of healthy drinking-BMI-sleep (OR: 0.42; 95% CI: 0.26 to 0.66).

**Conclusion:**

This cross-sectional study suggests that smoking, drinking, BMI, and sleep are significantly related with periodontitis and smoking is the principal risk factor related among them. This study provides various customized lifestyle combinations for periodontitis prevention.

**Supplementary Information:**

The online version contains supplementary material available at 10.1186/s12903-024-03937-z.

## Background

Periodontitis is a chronic inflammatory disease characterized by inflammation and progressive destruction of the supportive tissues surrounding teeth [1]. According to the National Health and Nutrition Examination Survey (NHANES), it is the second largest oral health problem and the sixth most common human disease worldwide, affecting approximately 46% of adults in the United States [2]. International studies have indicated that severe periodontal diseases could cause high-cost global burden with an annual expenditure of about US$ 82 billion [3]. As the primary reason for tooth loss in adults, periodontitis has a detrimental effect on masticatory dysfunction, self-esteem and oral health-related quality of life (OHRQoL) [4]. A growing body of literature has examined that periodontitis can influence chronic systemic health outcomes by increasing the risk for cardiovascular disease (CVD), type 2 diabetes mellitus (T2DM), and Alzheimer’s disease (AD) [1, 5, 6]. Recent animal model studies have elucidated the biological mechanisms based on the imbalance between periodontal microbiome and host immune response [1, 7]. For all these reasons, it is necessary to explore preventive measures for periodontitis to promote overall health and further contribute to chronic systemic diseases control.

In recent years, healthy lifestyles, as modifiable and low-cost behavioral factors, have attracted increasing attention and concern due to their potential health-related benefits for overall well-being [7–9]. Healthy lifestyle pattern can be determined by cigarette smoking (never smoking), physical activity (≥ 3.5 h/week moderate to vigorous intensity activity), high diet quality (upper 40% of Alternate Healthy Eating Index), moderate alcohol intake of 5–15 g/day (women) or 5–30 g/day (men), and normal weight (body mass index 18.5–24.9) on the basis of cardiovascular risk [8]. Meanwhile, individual lifestyle variable emerged as a complex network of interacting effects, not a single entity, or even a single static factor, and large heterogeneity existed between individual lifestyles [9, 10]. Nowadays, researches are focusing on the association between comprehensive healthy lifestyle pattern and chronic systemic diseases based on several large population-based cross sectional and cohort studies [8, 10, 11]. Similarly, both conventional and emerging lifestyles combinations have been reported to be associated with life expectancy [10], cognitive function [12], cardiovascular diseases [13]. As periodontitis and chronic systemic diseases are closely connected by similar epidemiological risk factors and biological mechanisms, a potential link may be achieved by chronic systemic healthy lifestyles to periodontitis prevention.

Periodontitis has been reported to relate to the interaction of multiple environmental, genetic and epigenetic factors, but the effect of primary prevention for periodontitis by healthy lifestyle combinations is often overlooked [1, 6]. Previous studies mainly focused on the effect of single lifestyle component on periodontitis [14–18] and of these, smoking and drinking received much greater attention, or even their interactive effect on periodontitis [14]. This is because these two common health risk behaviors served as important factors for the excess risk of periodontitis and they frequently co-occurred, which were well supported by epidemiological, etiological, and molecular evidence [14, 19]. Furthermore, previous research has extensively examined other conventional modifiable lifestyle factors, including maintaining a healthy BMI [15], adhering to healthy dietary intake [17], and engaging in regular physical activity [18]. However, recent studies have also explored new emerging lifestyle factors, such as sufficient sleep duration [16] and less sedentary behavior [20]. While numerous studies have been conducted to explore the correlation between conventional lifestyles and periodontitis rates, the evidence of the integration of conventional and emerging lifestyles is lacking, especially in population with periodontitis. In addition, previous findings were hindered by limitations in both sample size and study content, resulting in unclear and equivocal results despite previous extensive investigations into various lifestyle factors. It is worth noting that the modification of health behaviors should be preferred to circumvent the severe side effects of medical or surgical treatments, thereby offering cost-effective strategies for preventing or delaying periodontitis and its related complications. Therefore, further studies are needed to determine the relationship between periodontitis and comprehensive healthy lifestyle evaluation. In contrast to prior investigations, the current study aims to not only establish a comprehensive scoring system but also investigate the optimal combination of cost-effective lifestyle interventions and the utilization of conventional and emerging lifestyle information to identify at-risk groups [10, 19].

To fill this knowledge gap, based on the US National Health and Nutrition Examination Survey (NHANES 2009–2014), we aimed to develop a comprehensive lifestyle risk scoring system to investigate the correlation between periodontitis and lifestyle risk factors as a combined concept. Then, we identified the optimal lifestyle combinations from the random assemblage pattern for periodontitis prevention.

## Methods

### Study design and populations

**Fig. 1:**
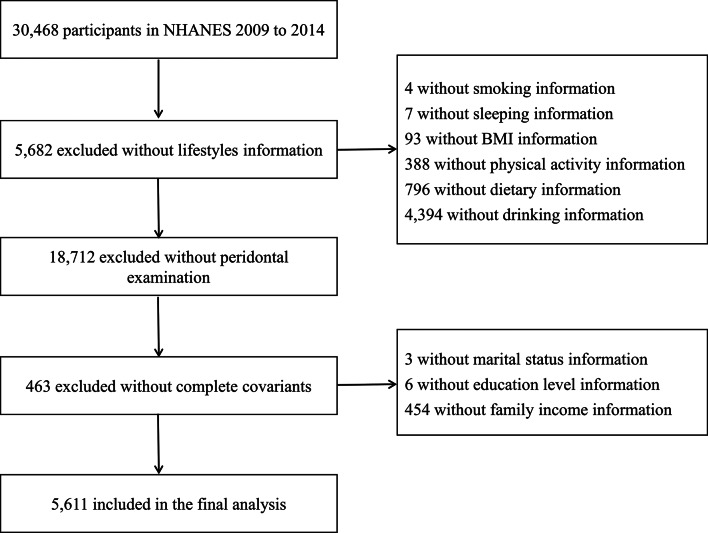
Data analysis screening flow chart

A total of 5,611 individuals were included in this study for the analysis using NHANES 2009–2014 data (Fig. 1). Participants were invited to complete oral examination and health examination in the mobile examination centers (MECs). Based on a computerized 24-hour dietary recall method, the first dietary measurement was administered via a face-to-face interview and the second was collected by telephone. Age, gender, ethnicity, educational level, smoking and drinking habits, sleep patterns, physical activity and medical history were collected by self-reported questionnaires. All NHANES protocols that generated the data used were approved by the National Center for Health Statistics (NCHS) at the Centers for Disease Control and Prevention (CDC).

### Lifestyle behaviors assessments

Detailed information on lifestyles was obtained through self-reported questionnaires. Each category of six healthy lifestyles was assigned a point based on specific criteria [21]. These criteria included non-smoking, defined as having smoked fewer than 100 cigarettes in their lifetime, and low-to-moderate alcohol drinking, which was determined by a daily consumption of one drink or fewer for females and two drinks or fewer for males [13]. The dietary guidelines in the US were used as a reference, with one drink containing 14 g of ethanol [13]. Additional criteria that were taken into account included ensuring sufficient sleep duration, ranging from 7 to 9 h [22], as well as maintaining a moderate body weight indicated by a body mass index (BMI) between 18.5 and 25.0 [11]. Furthermore, adequate physical activity (PA) was also considered, which was determined by the metabolic equivalent of task (MET) values ranging from 500 to 2000 [23, 24]. Lastly, appropriate energy intake was assessed using total energy intake measurements, which were set at 2400 to 3000 kcal for males and 1800 to 2400 kcal for females [25]. The healthy lifestyle total scores were finally summarized by the number of healthy lifestyle factors, ranging from 0 (lowest healthy score, highest risk) to 6 (highest healthy score, lowest risk). Then, the total scores can be categorized into three levels: optimal (4–6 points), intermediate (2–3 points), or poor (0–1 point).

### Outcomes

According to the NHANES (2009 to 2014) oral health data sets, periodontal probing depth (PD) and clinical attachment loss (CAL) measurements were taken at six sites per tooth (28 teeth without wisdom teeth) by certified examiners. A maximum of 168 sites and 28 teeth per participant could be examined to evaluate periodontal status. The identification and classification of periodontitis were based on the 2018 world classification [26]. Periodontitis diagnosis was determined by the presence of interdental CAL (clinical attachment loss) of at least 1 mm at two or more non-adjacent teeth, or buccal or oral CAL of at least 3 mm with pocketing depth greater than 3 mm at two or more teeth. The staging of periodontitis was defined as follows: a CAL of 1–2 mm was classified as stage I, a CAL of 3–4 mm as stage II, and a CAL of 5 mm or greater as stage III/IV. Moreover, to consider the complexity of management, patients initially diagnosed with Stage II periodontitis were reclassified as Stage III if their maximum PD (probing depth) measurement was 6 mm or greater. Additionally, patients diagnosed with Stage III periodontitis were reclassified as Stage IV if their teeth number was below 20 (10 pairs) [27].

### Statistical analyses

Considering the complicated sample design, all analyses accounted for the NHANES sampling weights to provide representative data for the American population. Descriptive data were shown as mean (SD) while categorical variables were reported as n (%). Chi square (χ2) tests and analysis of variance (ANOVA) were used to compare the distribution of baseline demographic characteristics among different lifestyle score groups.

Generalized linear regression models (GLM) and restricted cubic splines (RCS) were used to analyze the association between the single lifestyle factor and periodontitis. All covariates included age, gender, ethnicity, educational, marital status, history of diabetes and self-reported oral health, family income-to-poverty ratio (determined by comparing household income to the federal poverty guidelines of the U.S. Department of Health and Human Services). Three models were implemented for excluding the influence of covariates: Model 1 was an initial model without covariates; Model 2 was adjusted for age and gender; Model 3 was adjusted for ethnicity, family income-to-poverty ratio, educational level, and history of diabetes on the base of Model 2. Odds ratio (OR) and 95% confidence interval (CI) were calculated.

Then, the relationship was established between the total healthy lifestyle score and periodontitis outcome (present or absent) and sensitivity analyses was further performed for each distinct periodontitis group (Stage I/II, Stage III/IV) using the adjusted logistic regression model. In addition, linear regression model was further used to explore the relationship between the scoring of healthy lifestyle factors and the staging of periodontitis, as indicated by the clinical measurement of CAL. The subgroup analysis was further performed to investigate the association regarding demographic and clinical features. Based on the above lifestyle factors with major effects, different healthy lifestyle combinations were examined so that 16 combination groups were included in random assemblage. Only those combinations (number = 10) with exposure sample size ≥ 100 were taken into consideration. The statistical analyses were performed using SPSS version 26 (Statistical Package for the Social Sciences, IBM, Armonk, New York) and R version 4.3.1. A two-sided *p*-value of < 0.05 was considered statistically significant.

## Results

### Demographic and periodontal characteristics

Baseline characteristics by each lifestyle score group were presented in Table 1. There were 5,611 participants included in this study: 1,815 participants with 0–1 healthy lifestyle factor (mean age 52.12 (13.51)), 3,233 with 2–3 healthy lifestyle factors (mean age 52.33 (14.38)), and 563 with 4–6 healthy lifestyle factors (mean age 50.63 (14.82)). Four lifestyle factors, including non-smoking, moderate alcohol consumption, healthy BMI, and healthy sleep, had significant difference between the periodontitis and healthy groups (*p* < 0.05, Table 2).

**Table 1:** Demographic baseline characteristics: NHANES (2009 to 2014)

Characteristics	Healthy lifestyle score				
	All (*n*=5611)	0-1 (*n*=1815)	2-3 (*n*=3233)	4-6 (*n*=563)	p value
**Age (years), Mean** (**SD)**	52.09 (14.16)	52.12 (13.51)	52.33 (14.38)	50.63 (14.82)	0.03
**Gender, n (%)**					<0.01
Male	2935 (52.31)	871(47.99)	1731 (53.54)	333 (59.51)	
Female	2676 (47.69)	944 (52.01)	1502 (46.46)	230 (20.85)	
**Ethnicity, n (%)**					<0.01
Mexican American	666 (11.87)	242 (13.33)	380 (11.75)	44 (7.82)	
Other Hispanic	495 (8.82)	191(10.52)	267 (8.26)	37(6.57)	
Non-Hispanic White	2849 (50.78)	862 (47.49)	1668 (51.59)	319 (56.66)	
Non-Hispanic Black	1050 (18.71)	417 (22.98)	582 (18.00)	51 (9.06)	
Other Race-Including Multi-Racial	551 (9.82)	103 (5.67)	336 (10.39)	112 (19.89)	
**Marital status, n (%)**					<0.01
Married/Living with Partner	3690 (65.76)	1070 (58.95)	2205 (68.20)	415 (73.71)	
Widowed/Divorced/Separated/Never married	1921 (34.24)	745 (41.05)	1028 (31.80)	148 (26.29)	
**Educational level, n (%)**					<0.01
Less than high school	1013 (18.05)	428 (23.58)	537 (16.61)	48 (8.53)	
High school or higher	4589 (81.95)	1387 (76.42)	2696 (83.39)	515 (91.47)	
**Family income-to-poverty ratio, n (%)**					
Low income (≤1.85)	1998 (35.61)	821 (45.23)	1054 (32.60)	123 (21.85)	<0.01
High income (>1.85)	3613 (64.39)	994 (54.77)	2179 (67.40)	440 (78.15)	
**Smoking status, n (%)**					
No smoking	2881 (51.35)	396 (21.82)	1991 (61.58)	494 (87.74)	<0.01
Former smoking	1583 (28.21)	772 (42.53)	770 (23.82)	41 (7.28)	
Current smoking	1147 (20.44)	647 (35.65)	472 (14.60)	28 (4.97)	
**Drinking status, n (%)**					<0.01
0-14 g/d	1008 (17.96)	98 (5.40)	667 (20.63)	243 (43.16)	
14-56 g/d	3884 (69.22)	1362 (75.04)	2218 (68.61)	304 (54.00)	
>56 g/d	719 (12.81)	355 (19.56)	348 (10.76)	16 (2.84)	
**Sleeping status, n (%)**					<0.01
0-6 h	2146 (38.25)	1213 (66.83)	895 (27.68)	38 (6.75)	
7-9 h	3093 (55.12)	477 (26.28)	2128 (65.82)	488 (86.68)	
>9 h	372 (6.63)	125 (6.89)	210 (6.50)	37 (6.57)	
**BMI status, n (%)**					<0.01
Underweight (18.5 kg/m^2^)	63 (1.12)	33 (1.82)	27 (0.84)	3 (0.53)	
Normal (18.5-30 kg/m^2^)	3488 (62.16)	895 (49.31)	2106 (65.14)	487 (86.50)	
Obese (>30 kg/m^2^)	2060 (36.71)	887 (48.87)	1100 (34.02)	73 (12.97)	
**Physical activity level, n (%)**					<0.01
Lower 1/3 MET	2521 (44.93)	1042 (57.41)	1361 (42.10)	118 (20.96)	
Middle 1/3 MET	1843 (32.85)	280 (15.43)	1176 (36.37)	387 (68.74)	
Upper 1/3 MET	1247 (22.22)	493 (27.16)	696 (21.53)	58 (10.30)	
**Energy intake level, n (%)**					<0.01
Insufficient intake	2441 (43.50)	864 (47.60)	1380 (42.68)	197 (34.99)	
Adequate intake	2513 (44.79)	878 (48.37)	1426 (44.11)	209 (37.12)	
Excess intake	657 (11.71)	73 (4.02)	427 (13.21)	157 (27.89)	
**History of diabetes, n (%)**					<0.01
No	4858 (86.58)	1507 (83.03)	2829 (87.50)	522 (92.72)	
Yes	633 (11.28)	258 (14.21)	340 (10.52)	35 (6.22)	
Unknown or missing	120 (2.14)	50 (2.75)	64 (1.98)	6 (1.07)	
**Self-reported oral health, n (%)**					<0.01
Excellent/Very good/Good	3930 (70.04)	1104 (60.83)	2359 (72.97)	469 (82.95)	
Fair/Poor	1610 (28.69)	681 (37.52)	835 (25.83)	94 (16.07)	
Unknown or missing	71 (1.27)	30 (1.65)	39 (1.21)	2 (0.36)	

Descriptive data were shown as mean (SD) while categorical variables were reported as n (%). P-values less than 0.05 (*p* < 0.05) were considered significant. n: number

**Table 2:** Differences between periodontitis and healthy groups regarding lifestyles characteristics

	Healthy Control (*n* = 2273)	Periodontitis (*n* = 3338)	p value
**Age (years), Mean** (**SD)**	49.14 (14.03)	54.11 (13.89)	< 0.01
**Gender, n (%)**			< 0.01
Male	968 (42.59)	1967 (58.93)	
Female	1305 (57.41)	1371 (41.07)	
**Ethnicity, n (%)**			< 0.01
Mexican American	192 (8.45)	474 (14.20)	
Other Hispanic	193 (8.49)	302 (9.05)	
Non-Hispanic White	1334 (58.69)	1515 (45.39)	
Non-Hispanic Black	364 (16.01)	686 (20.55)	
Other Race-Including Multi-Racial	190 (8.36)	361 (10.81)	
**Marital status, n (%)**			0.13
Married/Living with Partner	1521 (66.92)	2169 (64.98)	
Widowed/Divorced/Separated/Never married	752 (33.08)	1169 (35.02)	
**Educational level, n (%)**			< 0.01
Less than high school	310 (13.64)	703 (21.06)	
High school or higher	1963 (86.36)	2635 (78.94)	
**Family income-to-poverty ratio, n (%)**			< 0.01
Low income (≤ 1.85)	681 (29.96)	1317 (39.45)	
High income (> 1.85)	1592 (70.04)	2021 (60.55)	
**History of diabetes, n (%)**			< 0.01
No	2012 (88.52)	2846 (85.26)	
Yes	215 (9.46)	418 (12.52)	
Unknown or missing	46 (2.02)	74 (2.22)	
**Self-reported oral health, n (%)**			< 0.01
Excellent/Very good/Good	1821 (80.11)	2109 (63.18)	
Fair/Poor	391 (17.20)	1219 (36.52)	
Unknown or missing	61(2.68)	10 (0.30)	
**No Smoking, n (%)**			< 0.01
No	948 (41.71)	1782 (53.39)	
Yes	1325 (58.29)	1556 (46.61)	
**Healthy drinking, n (%)**			0.01
No	188 (8.27)	353 (10.58)	
Yes	2085 (91.73)	2985 (89.42)	
**Healthy sleeping, n (%)**			< 0.01
No	858 (37.75)	1403 (42.03)	
Yes	1415 (62.25)	1935 (57.97)	
**Healthy BMI, n (%)**			< 0.01
No	1615 (71.05)	2515 (75.34)	
Yes	658 (28.95)	823 (24.66)	
**Healthy energy intake, n (%)**			0.90
No	2005 (88.21)	2948 (88.32)	
Yes	268 (11.79)	390 (11.68)	
**Healthy physical activities, n (%)**			0.38
No	1754 (77.17)	2609 (78.16)	
Yes	519 (22.83)	729 (21.84)	

Descriptive data were shown as mean (SD) while categorical variables were reported as n (%). P-values less than 0.05 (*p* < 0.05) were considered significant. N: number

### Associations of single lifestyle factor with periodontitis

**Fig. 2:**
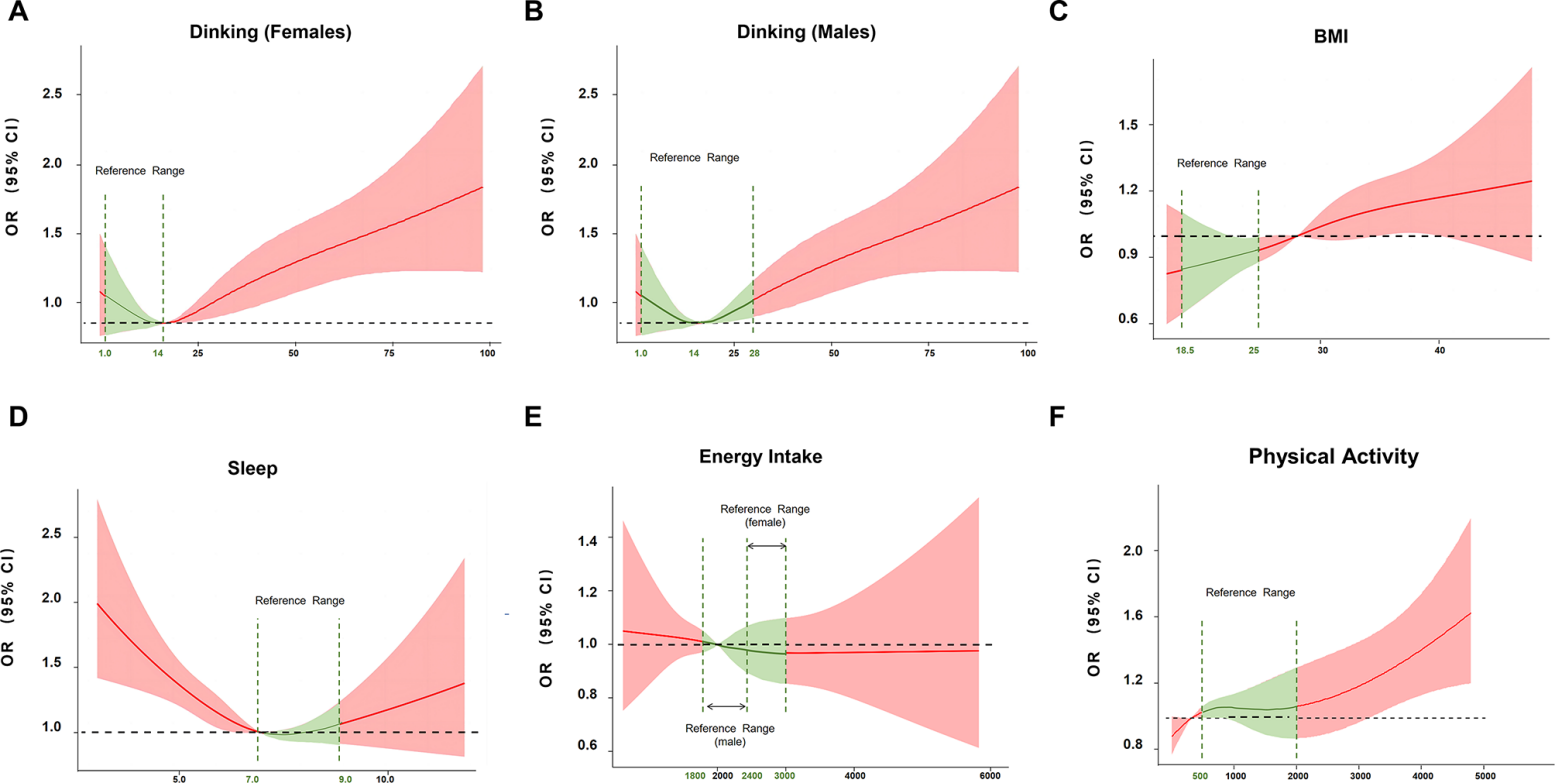
The restricted cubic spline for the association between single lifestyle factor and periodontitis. **A**) Drinking for females; **B**) Drinking for males; **C**) BMI; **D**) Sleep; **E**) Energy Intake; **F**) Physical Activity. *P*-values less than 0.05 (*p* < 0.05) were considered significant. Odds ratios and 95% CI for lifestyles in multivariate logistic regression models for periodontitis. BMI: body mass index, OR: odds ratios, CI: confidence interval

Results showed that the prevalence of periodontitis was the highest in current smokers (OR: 2.22, 95% CI: 1.82 to 2.71), followed by former smokers (OR: 1.36, 95% CI: 1.14 to 1.64) and non-smokers (Figure S1). The restricted cubic splines model revealed the non-linear relationship between the prevalence of periodontitis and alcohol consumption quantity (*p*_Drinking−Female_ for nonlinearity = 0.006, Fig. 2A; *p*_Drinking−Male_ = 0.047, Fig. 2B). Furthermore, the logistic regression model indicated that heavy drinking (> 56 g/d) significantly increased periodontitis prevalence (OR: 1.51, 95% CI: 1.14 to 1.99), however, no statistical difference (OR: 0.96, 95% CI: 0.81 to 1.14) was identified between the association of moderate drinking (14–56 g/d) and periodontitis prevalence (Figure S1). Moreover, obese (BMI ≥ 30) (OR: 1.38, 95% CI: 1.16 to 1.64) and overweight populations (25 ≤ BMI < 30) (OR: 1.38, 95% CI: 1.17 to 1.62) had a higher periodontitis prevalence than normal weight populations (18.5 ≤ BMI < 25.0) (Figure S1). Regarding sleep duration, the non-linear dose-response relationship was found between periodontitis prevalence and sleep duration (*p*_Sleep_ for nonlinearity = 0.004) (Fig. 2D). Additionally, the logistic regression model revealed that sleep deprivation (< 7 h) had a higher periodontitis prevalence than their counterparts (7–9 h) (OR: 1.28, 95% CI: 1.08 to 1.53) (Figure S1). For energy intake and physical exercise, insufficient intake (OR: 1.17, 95% CI: 1.01 to 1.35) and overactivity (OR: 1.21, 95% CI: 1.01 to 1.45) was found associated with an increased prevalence of periodontitis in the initial model (Figure S1). However, we found no evidence of nonlinearity in associations between the prevalence of periodontitis and lifestyles including BMI, energy intake and physical activity (*p* values > 0.05, Fig. 2C, E, F).

### Associations of comprehensive lifestyle lactors with periodontitis

Compared with individuals with 0–1 healthy lifestyle factor, the periodontitis prevalence was lower among those with 2–3 healthy lifestyle factors (OR: 0.83, 95% CI: 0.71 to 0.98) and 4–6 healthy lifestyle factors (OR: 0.66, 95% CI: 0.50 to 0.87) in the total population when compared with the normal group (Table 3). Each additional healthy lifestyle was associated with 11% lower prevalence for periodontitis (OR: 0.89, 95% CI: 0.83 to 0.96). In the sensitivity analysis, compared with stage I/II periodontitis, each additional factor were significantly associated lower prevalence with stage III/IV periodontitis, as shown in Table S1 (model 1: OR: 0.81, 95% CI: 0.76 to 0.86; model 2: OR: 0.77, 95% CI: 0.72 to 0.82; model 3: OR: 0.88, 95% CI: 0.72 to 0.94). Furthermore, to further explore the potential relationship between lifestyle variables and different stages of periodontitis, we established a linear regression model and identified the negative regression association between healthy lifestyle factors and clinical attachment loss of the severe site in the group with periodontitis. In addition, the association between periodontitis and lifestyles had statistically significant differences regarding sociodemographic characteristics, as demonstrated by subgroup analyses (Tables 1, 2 and 3). When populations were risk stratified, results showed that adherence to 4–6 healthy lifestyles significantly reduced the prevalence of periodontitis in most groups (except for elderly and low-income groups), while for females (OR: 0.75, 95% CI: 0.60 to 0.95) and relatively high-income populations (OR: 0.74, 95% CI: 0.60 to 0.91), they could benefit from adherence to only 2–3 healthy lifestyles (Table 3).

**Table 3:** Periodontitis Risk by lifestyle factors scoring

		0–1 healthy lifestyle factor	2–3 healthy lifestyle factors			4–6 healthy lifestyle factors			Each additional healthy lifestyle factor		
			OR	95% CI	p	OR	95% CI	p	OR	95% CI	p
**Total**	Model 1	Ref	0.80	(0.70, 0.91)	< 0.01	0.53	(0.42, 0.67)	< 0.01	0.84	(0.80, 0.89)	< 0.01
	Model 2	Ref	0.74	(0.64, 0.85)	< 0.01	0.50	(0.38, 0.65)	< 0.01	0.82	(0.77, 0.88)	< 0.01
	Model 3	Ref	0.83	(0.71, 0.98)	0.03	0.66	(0.50, 0.87)	< 0.01	0.89	(0.83, 0.96)	< 0.01
**Age**											
< 65	Model 1	Ref	0.71	(0.61, 0.81)	< 0.01	0.42	(0.32, 0.57)	< 0.01	0.78	(0.73, 0.84)	< 0.01
	Model 2	Ref	0.67	(0.57, 0.79)	< 0.01	0.41	(0.29, 0.56)	< 0.01	0.77	(0.72, 0.83)	< 0.01
	Model 3	Ref	0.79	(0.66, 0.95)	0.01	0.60	(0.43, 0.83)	< 0.01	0.86	(0.79, 0.93)	< 0.01
≥ 65	Model 1	Ref	1.34	(0.96, 1.86)	0.08	1.75	(0.99, 3.09)	0.06	1.25	(1.10, 1.42)	< 0.01
	Model 2	Ref	1.30	(0.93, 1.83)	0.12	1.64	(0.91, 2.95)	0.10	1.23	(1.08, 1.40)	< 0.01
	Model 3	Ref	1.23	(0.86, 1.77)	0.25	1.48	(0.80, 2.76)	0.21	1.19	(1.03, 1.38)	0.02
**Gender**											
Male	Model 1	Ref	0.86	(0.70, 1.07)	0.18	0.51	(0.37, 0.70)	< 0.01	0.84	(0.77, 0.92)	< 0.01
	Model 2	Ref	0.82	(0.67, 1.02)	0.08	0.50	(0.37, 0.69)	< 0.01	0.84	(0.77, 0.91)	< 0.01
	Model 3	Ref	0.92	(0.73, 1.18)	0.52	0.65	(0.46, 0.91)	0.01	0.90	(0.82, 0.99)	0.03
Female	Model 1	Ref	0.69	(0.57, 0.85)	< 0.01	0.49	(0.33, 0.73)	< 0.01	0.81	(0.74, 0.88)	< 0.01
	Model 2	Ref	0.67	(0.54, 0.83)	< 0.01	0.51	(0.34, 0.76)	< 0.01	0.81	(0.74, 0.89)	< 0.01
	Model 3	Ref	0.75	(0.60, 0.95)	0.02	0.69	(0.45, 1.05)	0.08	0.88	(0.79, 0.98)	0.02
**Education level**											
Less than high school	Model 1	Ref	0.82	(0.60, 1.11)	0.19	0.43	(0.20, 0.92)	0.03	0.86	(0.74, 1.00)	0.05
	Model 2	Ref	0.76	(0.55, 1.06)	0.10	0.35	(0.15, 0.80)	0.01	0.83	(0.70, 0.98)	0.03
	Model 3	Ref	0.70	(0.50, 0.98)	0.04	0.29	(0.13, 0.65)	< 0.01	0.79	(0.66, 0.94)	< 0.01
High school or higher	Model 1	Ref	0.83	(0.72, 0.96)	0.01	0.58	(0.46, 0.74)	< 0.01	0.86	(0.81, 0.92)	< 0.01
	Model 2	Ref	0.76	(0.65, 0.90)	< 0.01	0.54	(0.41, 0.72)	< 0.01	0.84	(0.79, 0.90)	< 0.01
	Model 3	Ref	0.86	(0.72, 1.02)	0.09	0.69	(0.51, 0.93)	0.01	0.90	(0.84, 0.98)	0.01
**Marital status**											
Married/Living with Partner	Model 1	Ref	0.75	(0.65, 0.87)	< 0.01	0.49	(0.36, 0.67)	< 0.01	0.82	(0.75, 0.89)	< 0.01
	Model 2	Ref	0.69	(0.59, 0.82)	< 0.01	0.47	(0.34, 0.64)	< 0.01	0.80	(0.74, 0.88)	< 0.01
	Model 3	Ref	0.80	(0.65, 0.98)	0.03	0.65	(0.44, 0.95)	0.03	0.89	(0.80, 0.98)	0.02
Widowed/Divorced/Separated/Never married	Model 1	Ref	0.97	(0.76, 1.24)	0.82	0.78	(0.45, 1.34)	0.36	0.97	(0.86, 1.08)	0.53
	Model 2	Ref	0.91	(0.70, 1.19)	0.47	0.73	(0.41, 1.29)	0.27	0.93	(0.83, 1.05)	0.22
	Model 3	Ref	0.93	(0.71, 1.22)	0.60	0.77	(0.42, 1.41)	0.39	0.95	(0.83, 1.08)	0.42
**Family income-to-poverty ratio**											
Low income (≤ 1.85)	Model 1	Ref	1.21	(0.98, 1.50)	0.08	0.96	(0.56, 1.67)	0.89	1.02	(0.91, 1.15)	0.71
	Model 2	Ref	1.14	(0.93, 1.41)	0.21	0.79	(0.46, 1.35)	0.38	0.98	(0.87, 1.09)	0.67
	Model 3	Ref	1.13	(0.92, 1.39)	0.24	0.86	(0.50, 1.46)	0.56	0.98	(0.87, 1.10)	0.75
High income (> 1.85)	Model 1	Ref	0.74	(0.63, 0.88)	< 0.01	0.51	(0.38, 0.69)	< 0.01	0.83	(0.77, 0.90)	< 0.01
	Model 2	Ref	0.69	(0.57, 0.83)	< 0.01	0.50	(0.36, 0.70)	< 0.01	0.83	(0.76, 0.90)	< 0.01
	Model 3	Ref	0.74	(0.60, 0.91)	< 0.01	0.60	(0.43, 0.84)	< 0.01	0.87	(0.80, 0.95)	< 0.01

No covariates were adjusted in Model 1. Model 2 was adjusted for age and gender. Model 3 was adjusted for ethnicity, family income-to-poverty ratio, educational level, and history of diabetes on the base of Model 2. P-values less than 0.05 (*p* < 0.05) were considered significant. OR: odds ratio; CI: confidence interval

### Identification of optimal lifestyle combinations

**Fig. 3:**
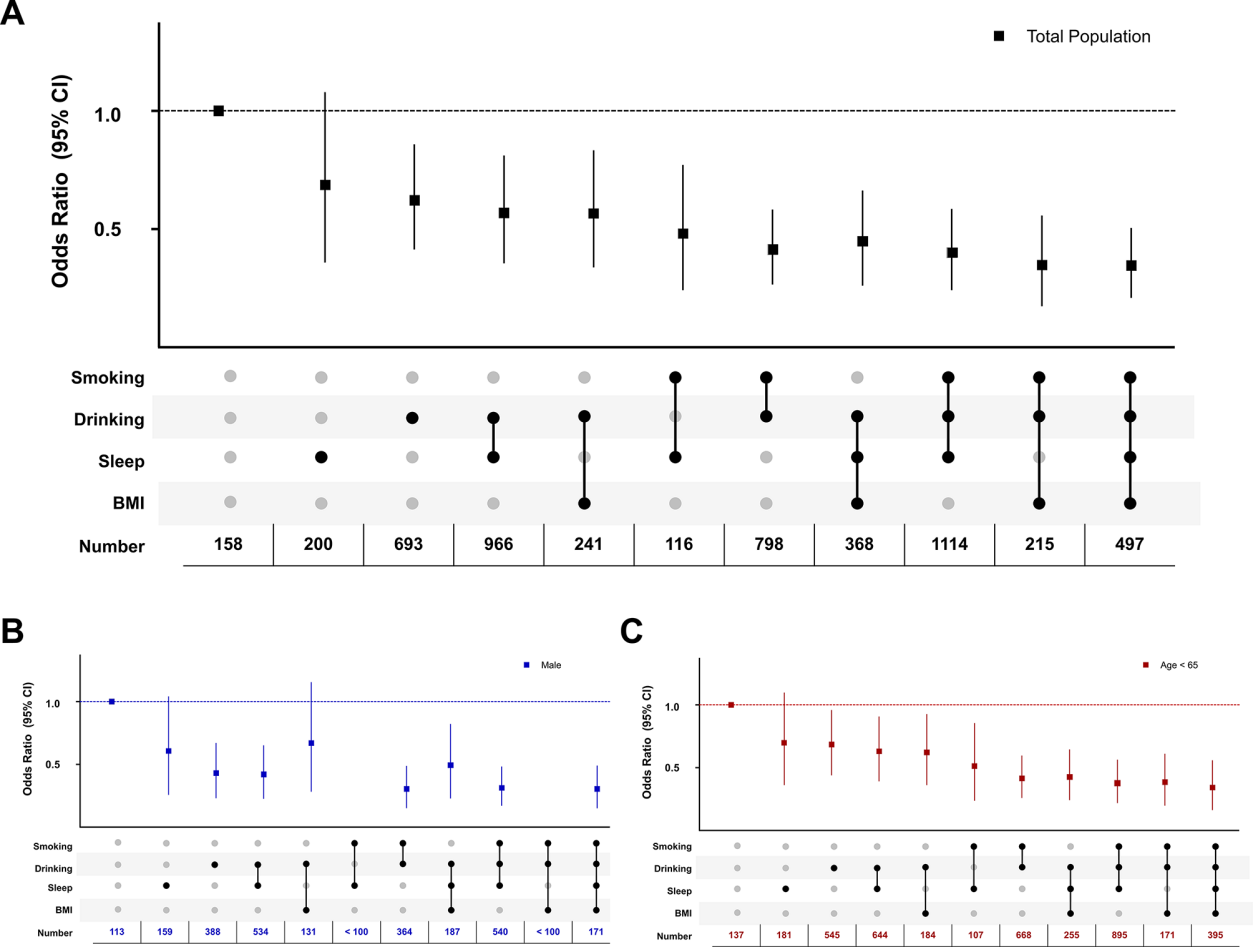
Association between healthy lifestyle combinations and the prevalence of periodontitis. **A**) OR in the total population; **B**) OR in males; **C**) OR in populations with age < 65 years. *P*-values less than 0.05 (*p* < 0.05) were considered significant. OR: odds ratio, CI: confidence interval, n: number

Through random combinations, results showed that the combination of four lifestyle factors (smoking, drinking, sleep, and BMI) had the lowest periodontitis prevalence (OR: 0.32, 95% CI: 0.21 to 0.50). For three lifestyle combinations, however, not all of them outperformed the two combinations models; the two-combination of smoking-drinking (OR: 0.39, 95% CI: 0.27 to 0.58) had relatively lower prevalence than the three-combination of healthy drinking-BMI-sleep (OR: 0.42, 95% CI: 0.26 to 0.66) (Fig. 3A) in the total population. In the sensitivity analysis, the similar significant results were identified in males (OR: 0.36, 95% CI: 0.13 to 0.96) and populations aged less than 65 years (OR: 0.35, 95% CI: 0.20 to 0.61) (Fig. 3B, C; Table S3).

## Discussion

**Fig. 4:**
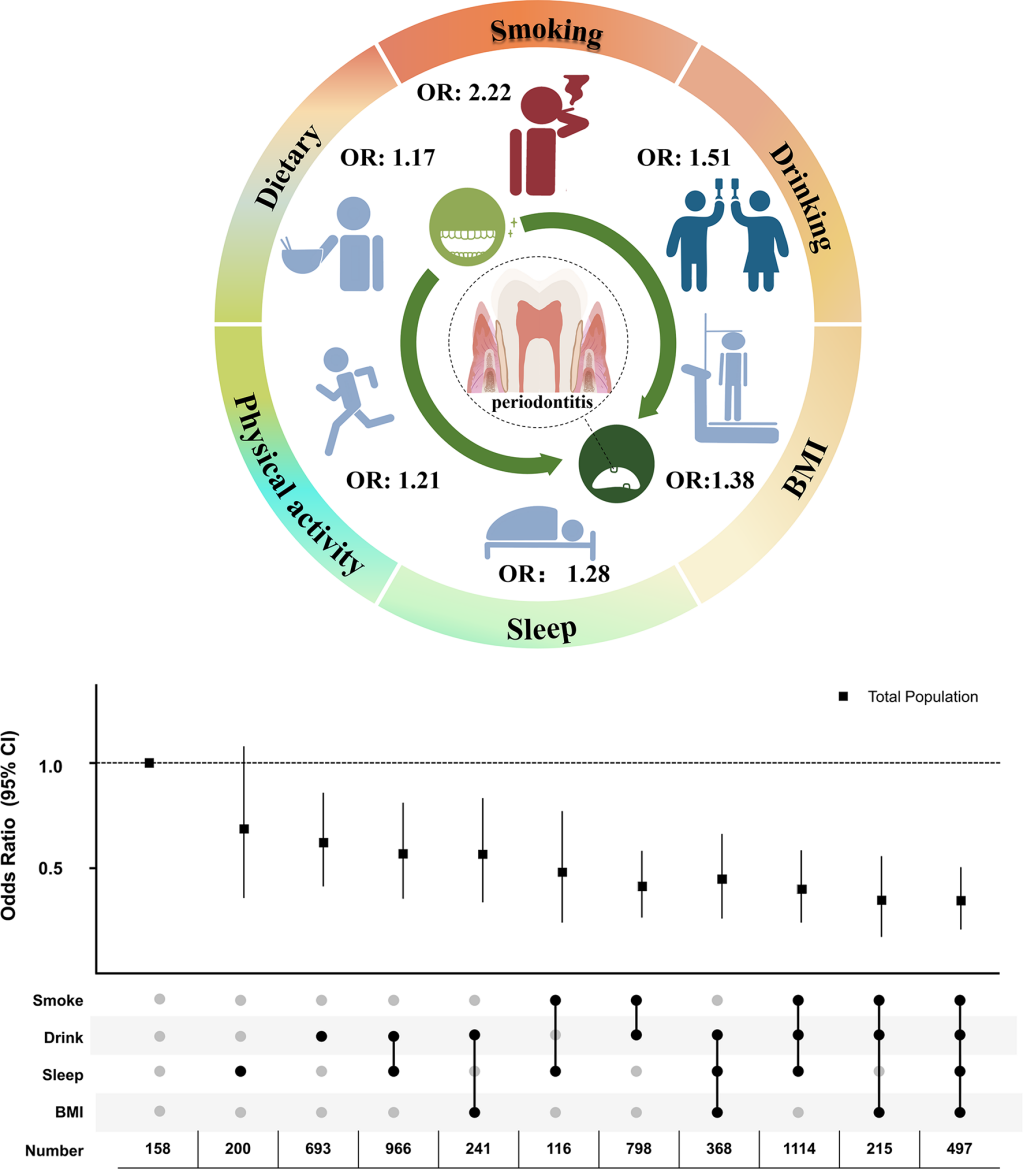
Independent and combined effects of lifestyles on the prevalence of periodontitis

This is the first chronic systemic evaluation of comprehensive lifestyle factors and different lifestyle combinations in periodontitis prevalence. First, the lifestyle scoring system was established based on six modifiable lifestyles factors which associated with periodontitis prevalence. Then, the four lifestyle factors (smoking, drinking, BMI, and sleep) were singled out as the most pronounced variables between the periodontitis and healthy groups. In line with this, the optimal lifestyle combinations were identified targeting periodontitis prevention. Results further showed that the combination of four healthy lifestyles had the lowest periodontitis prevalence compared with other combinations, whereas not all three lifestyle combinations outperformed two combinations models. Moreover, smoking was assessed as the main lifestyle factor contributing to increased periodontitis prevalence by multi-dimensional evaluation, and smokers could select more other modifiable lifestyles accompanied with lower periodontitis prevalence. This research provides the evidence-based medicine evidence for periodontitis prevention, like other chronic diseases, through modifiable lifestyles combinations (Fig. 4).

In our analysis, smoking and drinking served as the top two risk factors in periodontitis [14]. It is well acknowledged that smoking is not only a common environmental factor posing a health hazard [28] but also a strong risk factor for periodontal diseases, consistent with our results [14, 19]. This can be partially attributed to altered inflammatory response, microbial composition, and an impaired ability of periodontium resulting from cigarette smoke extracts [19]. By smoking habits, smoking can be further classified into former smoking and current smoking because the hazards of smoking on generating chronic systemic inflammation can continue for months or years despite smoking cessation [28, 29]. This was coincident with our results that the periodontitis prevalence was highest in current smokers, followed by former smokers and non-smokers. Unlike smoking, drinking and periodontitis have a paradoxical relationship, as demonstrated by previous studies, and there existed clear gender differences between them [30]. While there was one meta-analysis confirming the dose-independent detrimental effect between drinking and periodontitis [31], some researches have reservations [30]. The heterogeneity in these studies could be attributed to the quantity, frequency, timing, and pattern of alcohol consumption [32], as our restricted cubic spline model showed.

Additionally, adherence to other relatively niche lifestyles can further strengthen the health-enhancing effect of lifestyles. Incorporating previous studies, this study validated the nonlinear trend between periodontitis and BMI in a dose-dependent manner, in which the higher BMI, the higher periodontitis prevalence. Possible underlying mechanisms may be elevated inflammatory markers in periodontal pockets and altered bacterial profiles of saliva for obese populations [33]. On the other hand, sleep deprivation was reported to increase periodontitis risk and in line with our epidemiological evidence, animal models revealed the potential mechanism of sleep deprivation in increased alveolar bone loss [34]. Based on one previous systematic literature review, it was observed that there exists an inverse relationship between various dietary factors such as fatty acids, vitamin C, vitamin E, beta-carotene, fiber, calcium, dairy, fruits, and vegetables, and the likelihood of developing periodontal disease, suggesting that inadequate dietary intake is associated with an elevated risk of periodontal disease. Furthermore, our investigation into total energy intake corroborated and extended these findings, indicating that both the energy contribution and nutritional composition of the dietary can influence the condition of periodontal health [17]. Regarding physical activity, it is worth noting that the concept of exercise health has increased in popularity and our results have indicated the harmful effect of physical overactivity on oral health [20, 35]. Moreover, Pamela Shaw et al. have reported that physical exercise was indeed a risk factor for amyotrophic lateral sclerosis (ALS), indicating that relatively high exercise volume may induce early onset of diseases [35]. The possible underlying mechanism may be the abnormal inflammatory reaction and unstable blood glucose levels in response to overactivity, particularly when exercise volume exceeds the body tolerance [36]. This is the first time such a finding has been made with periodontitis; therefore, physical activity should not be viewed in a one-sided manner, and future studies should focus on where exercise interventions do and do not work well.

This is the first study to establish a lifestyle scoring system for periodontitis based on six low-risk modifiable lifestyles. These results showed that populations with the healthiest lifestyles had 34% decreased periodontitis prevalence compared with populations with the least-healthy lifestyles. Further results indicated that the prevalence of periodontitis can be reduced by 11% for each increment of 1 in the lifestyle score. Moreover, our study revealed a significant inverse correlation between the overall lifestyle score and the severity of periodontitis. Such similar health scoring system has been well established based on the large population data to raise life expectancy and prevent or delay chronic diseases. For example, the cohort study in the US population concluded that adhering to five low-risk lifestyle factors could increase life expectancy by 14.0 years for females and 12.2 years for males [11]. Similarly, a similar conclusion also could arise from this study that this health-enhancing effect on periodontitis changed with sociodemographic characteristics. The subgroup analysis indicated that the degree of periodontitis prevalence decline varied by age, gender, and household income. Taken together, the comprehensive lifestyle management has relatively better prevention efficiency compared with the single lifestyle factor, which may bring more health-related benefits not only for chronic systemic diseases but for periodontitis prevention. Since this healthy lifestyle scoring system has been proven effective for periodontitis and other diseases, the sample size needs to be expanded for more in-depth research in the future.

Lifestyle modification is a relatively simple treatment without drug or surgical side effects [7–9]. However, different factors do not contribute to periodontitis risk to the same extent and not each person has access to all healthy lifestyles. Meanwhile, combined with previous data, our results illustrated that most populations tended to adhere to either 2 or 3 lifestyle scores rather than six lifestyles, despite the lowest periodontitis prevalence associated with those six lifestyles among all combinations. Therefore, this study was the first to investigate all risk combinations using a comprehensive combinational approach and to identify the optimal lifestyles combination among the four significantly different lifestyles (smoking, drinking, BMI, and sleep). Based on the dynamically random combinations, the combination of four lifestyles was found to have the strongest beneficial effects on periodontitis prevention, whereas all three lifestyle combinations outperformed the two combinations models except one combination (drinking, sleep, and BMI) [37, 38]. These results indirectly demonstrated the strongest harmful effect of smoking on periodontitis, in agreement with our single-factor analysis. All above highlighted the need for promoting tobacco-control activity and enforcing policies and regulations prohibiting smoking in public. However, it is difficult to stop smoking habit once it has been established for smoking addiction populations. Our results may propose a compromise that this kind of high-risk group could be intervened with other modifiable lifestyles as soon as possible, particularly selecting those lifestyles that can be easier to adhere to. The same conclusion can also be applied to non-smokers, in which the more lifestyles, the lower periodontitis prevalence. Overall, this health-enhancing effect indicated that the selection of lifestyle combinations should be personalized but not generalized. Thus, individuals should choose the combination that best suits their own experience according to their actual situation for individualized disease prevention.

The main strengths of this study included a large-scale population, and a comprehensive lifestyle evaluation to determine the optimal combinations. However, this study is not without limitations. First, no causality can be inferred due to the cross-sectional nature of the data. Then, measurement errors and recall bias may be present due to the self-reported nature of lifestyle questionnaires, therefore, the content of future lifestyles evaluations needs to be more diverse and detailed. In addition, the evaluation criteria for lifestyles were restricted to the US population, and further validation of large population-based studies in different ethnicities is needed in future research. Nonetheless, the findings from this research could offer insight into the lifestyle prevention for individuals with periodontitis and will stimulate further research and discussion.

## Conclusion

Combined healthy lifestyles were associated with generally lower prevalence of periodontitis by incorporating smoking, drinking, total energy intake, physical activity, sleep patterns and BMI. Based on the effectiveness and validity of the lifestyle scoring system, this study emphasizes the critical need for multi-component lifestyle management in populations with periodontitis, and this can help enhance future policies and interventions for oral and chronic systemic health.

### Electronic supplementary material

Below is the link to the electronic supplementary material.

Table S1: Association between healthy lifestyle factors and disease risk in the Stage III/IV periodontitis group compared with the Stage I/II periodontitis group. No covariates were adjusted in Model 1. Model 2 was adjusted for age and gender. Model 3 was adjusted for ethnicity, family income-to-poverty ratio, educational level, and history of diabetes on the base of Model 2. P-values less than 0.05 (*p* < 0.05) were considered significant. OR: odds ratio; CI: confidence interval; BMI: body mass index.

Table S2: Periodontitis prevalence in each lifestyle combination (All combinations). Odds ratio and 95% CI were calculated. *P*-values less than 0.05 (*p* < 0.05) were considered significant. ^*^ Represented *p* < 0.05, ^**^ Represented *p* < 0.01. n: number, CI: confidence interval.

Table S3: Linear regression model between healthy lifestyle factors and clinical attachment loss of the heaviest site in periodontitis group. No covariates were adjusted in Model 1. Model 2 was adjusted for age and gender. Model 3 was adjusted for ethnicity, family income-to-poverty ratio, educational level, and history of diabetes on the base of Model 2. P-values less than 0.05 (*p* < 0.05) were considered significant. CAL: clinical attachment loss; CI: confidence interval

Figure S1: Logistic regression models for associating single lifestyle factor with periodontitis prevalence. No covariates were adjusted in Model 1(Black). Model 2 (Blue) was adjusted for age and gender. Model 3 (Red) was adjusted for ethnicity, family income-to-poverty ratio, educational level, and history of diabetes on the base of Model 2. Odds ratio and 95% CI were calculated. *P*-values less than 0.05 (*p* < 0.05) were considered significant. CI: confidence interval.

## Data Availability

The datasets generated and/or analyzed during the current study are not publicly available due to the institution regulations, but are available from the corresponding author on reasonable request.

## Abbreviations

NHANES: National Health and Nutrition Examination Survey
BMI: body mass index; MET:metabolic equivalent of task
U.S.: United States
OR: odds ratio
CI: confidence interval
OHRQoL: oral health-related quality of life
CVD: cardiovascular diseases
T2DM: type 2 diabetes mellitus
AD: Alzheimer’s disease
PA: physical activity
MEC: mobile examination centers
NCHS: National Center for Health Statistics
CDC: Centers for Disease Control and Prevention
PD: Periodontal probing depth
CAL: clinical attachment loss
SD: standard deviation
ANOVA: analysis of variance
