# Supplementary material for: Association between healthy lifestyle combinations and periodontitis in NHANES
Source: BMC Oral Health. 2024 Feb 4;24:182. doi: 10.1186/s12903-024-03937-z (PMC10840229; doi:10.1186/s12903-024-03937-z)
Supplement: Supplementary file 1 — Table S1: Association between healthy lifestyle factors and disease risk in the Stage III/IV periodontitis group compared with the Stage I/II periodontitis group. No covariates were adjusted in Model 1. Model 2 was adjusted for age and gender. Model 3 was adjusted for ethnicity, family income-to-poverty ratio, educational level, and history of diabetes on the base of Model 2. P-values less than 0.05 (p < 0.05) were considered significant. OR: odds ratio; CI: confidence interval; BMI: body mass index. [file 12903_2024_3937_MOESM1_ESM.docx]

**Table S1** Association between healthy lifestyle factors and disease risk in the Stage III/IV periodontitis group compared with the Stage I/II periodontitis group.

| **Stages III/IV**  **VS Stages I/II** | **Model 1** | | | **Model 2** | | | **Model 3** | | |
| --- | --- | --- | --- | --- | --- | --- | --- | --- | --- |
|  | **OR** | **95% CI** | ***p* value** | **OR** | **95% CI** | ***p* value** | **OR** | **95% CI** | ***p* value** |
| **Healthy lifestyle factors** |  |  |  |  |  |  |  |  |  |
| 0-1 factors | Ref | | | Ref | | | Ref | | |
| 2-3 factors | 0.69 | (0.59, 0.80) | <0.01 | 0.61 | (0.52, 0.71) | <0.01 | 0.74 | (0.62, 0.87) | <0.01 |
| 4-6 factors | 0.50 | (0.38, 0.66) | <0.01 | 0.46 | (0.34, 0.61) | <0.01 | 0.71 | (0.52, 0.97) | 0.03 |
| Each additional factor | 0.81 | (0.76, 0.86) | <0.01 | 0.77 | (0.72, 0.82) | <0.01 | 0.88 | (0.82, 0.94) | <0.01 |
| **Smoking** |  |  |  |  |  |  |  |  |  |
| Yes | Ref | | | Ref | | | Ref | | |
| No | 0.44 | (0.36, 0.53) | <0.01 | 0.48 | (0.39, 0.58) | <0.01 | 0.57 | (0.46, 0.71) | <0.01 |
| **Drinking** |  |  |  |  |  |  |  |  |  |
| More than moderate | Ref | | | Ref | | | Ref | | |
| Low-to-moderate | 1.05 | (0.88, 1.25) | 0.59 | 0.69 | (0.57, 0.83) | <0.01 | 0.78 | (0.65, 0.93) | <0.01 |
| **Sleep** |  |  |  |  |  |  |  |  |  |
| Inadequate | Ref | | | Ref | | | Ref | | |
| Adequate | 0.80 | (0.69, 0.93) | <0.01 | 0.74 | (0.63, 0.86) | <0.01 | 0.89 | (0.76, 1.04) | 0.15 |
| **BMI** |  |  |  |  |  |  |  |  |  |
| Immodest | Ref | | | Ref | | | Ref | | |
| Modest | 0.87 | (0.71, 1.05) | 0.15 | 0.96 | (0.77, 1.20) | 0.71 | 1.12 | (0.88, 1.42) | 0.35 |

No covariates were adjusted in Model 1. Model 2 was adjusted for age and gender. Model 3 was adjusted for ethnicity, family income-to-poverty ratio, educational level, and history of diabetes on the base of Model 2. P-values less than 0.05 (*p* < 0.05) were considered significant. OR: odds ratio; CI: confidence interval; BMI: body mass index.
