# Supplementary material for: Association between healthy lifestyle combinations and periodontitis in NHANES
Source: BMC Oral Health. 2024 Feb 4;24:182. doi: 10.1186/s12903-024-03937-z (PMC10840229; doi:10.1186/s12903-024-03937-z)
Supplement: Supplementary file 2 — Table S2: Periodontitis prevalence in each lifestyle combination (All combinations). Odds ratio and 95% CI were calculated. P-values less than 0.05 (p < 0.05) were considered significant. * Represented p < 0.05, ** Represented p < 0.01. n: number, CI: confidence interval. [file 12903_2024_3937_MOESM2_ESM.docx]

**Table S1** Periodontitis risk in each lifestyle combination (All combinations)

| **Group** | | | | **Total** | | **Male** | | **Female** | | **Age < 65** | | **Age > 65** | |
| --- | --- | --- | --- | --- | --- | --- | --- | --- | --- | --- | --- | --- | --- |
| Smoking Sleep Drink BMI | | | | **OR (95%CI)** | **N =**  **5611** | **OR (95%CI)** | **N =**  **2935** | **OR (95%CI)** | **N =**  **2676** | **OR (95%CI)** | **N =**  **4407** | **OR (95%CI)** | **N =**  **1204** |
| × | × | × | × | Ref | 158 (2.82) | Ref | 113 (3.85) | Ref | 45 (1.68) | Ref | 137 (3.11) | Ref | 21 (1.74) |
|  | √ |  |  | 0.62(0.36, 1.08) | 85 (1.51) | 0.69(0.34, 1.39) | 58 (1.98) | 0.47 (0.18, 1.26) | 27 (1.01) | 0.63 (0.36, 1.10) | 83 (1.88) | 0.52 (0.10, 2.88) | 2 (0.17) |
|  |  | √ |  | 0.60(0.41, 0.86) ** | 200 (3.56) | 0.52 (0.31, 0.89) * | 159 (5.42) | 0.65 (0.36, 1.15) | 41 (1.53) | 0.65 (0.44, 0.96) * | 181 (4.11) | 0.46 (0.12, 1.78) | 19 (1.58) |
|  |  |  | √ | 0.75(0.30,1.92) | 693 (12.35) | 0.72 (0.27, 1.87) | 388 (13.22) | 1.10 (0.16, 7.42) | 305 (11.40) | 0.70 (0.25, 1.92) | 545 (12.37) | 1.51 (0.07, 33.77) | 148 (12.29) |
| √ |  |  |  | 0.31(0.17, 0.57) ** | 48 (0.86) | 0.38 (0.19, 0.77) ** | 38 (1.29) | 0.18 (0.07, 0.52) ** | 10 (0.37) | 0.30 (0.16, 0.56) ** | 45 (1.02) | / | 3 (0.25) |
|  | √ | √ |  | 0.54(0.36, 0.81) ** | 116 (2.07) | 0.51 (0.30, 0.87) ** | 90 (3.07) | 0.52 (0.28, 0.96) * | 26 (0.97) | 0.60 (0.39, 0.91) * | 107 (2.43) | 0.59 (0.16, 2.19) | 9 (0.75) |
| √ |  |  | √ | 0.53(0.16, 1.73) | 798 (14.22) | 0.35 (0.08, 1.47) | 364 (12.40) | 1.26 (0.24, 6.65) | 434 (16.22) | 0.57 (0.16, 2.06) | 668 (15.16) | 0.23 (0.01, 4.07) | 130 (10.80) |
|  | √ |  | √ | 0.91(0.28,2.92) | 23 (0.41) | 1.68 (0.46,6.07) | 16 (0.55) | 0.49 (0.09,2.60) | 7 (0.26) | 1.16 (0.31,4.37) | 21 (0.48) | 0.23 (0.03,1.81) | 2 (0.17) |
|  |  | √ | √ | 0.53(0.34, 0.83) ** | 966 (17.22) | 0.76 (0.38, 1.54) | 534 (18.19) | 0.39 (0.20, 0.76) ** | 432 (16.14) | 0.58 (0.36, 0.93) * | 644 (14.61) | 0.59 (0.14, 2.47) | 322 (26.74) |
| √ | √ |  |  | 0.43(0.24, 0.77) ** | 56 (1.00) | 0.40 (0.20, 0.80) ** | 43 (1.47) | 0.54 (0.17, 1.73) | 13 (0.49) | 0.45 (0.24, 0.85) * | 47 (1.07) | 0.34 (0.05, 2.24) | 9 (0.75) |
| √ |  | √ |  | 0.39(0.27, 0.58) ** | 241 (4.30) | 0.36 (0.20, 0.65) ** | 131 (4.46) | 0.40 (0.22, 0.70) ** | 110 (4.11) | 0.39 (0.26, 0.60) ** | 184 (4.18) | 0.53 (0.14, 2.05) | 57 (4.73) |
|  | √ | √ | √ | 0.42(0.26,0.66) ** | 1114 (19.85) | 0.58 (0.30,1.10) | 540 (18.40) | 0.32 (0.15,0.67) ** | 574 (21.45) | 0.40 (0.24,0.65) ** | 895 (20.31) | 0.76 (0.19,3.07) | 219 (18.19) |
| √ | √ | √ |  | 0.38(0.24,0.58) ** | 33 (0.59) | 0.38 (0.23,0.64) ** | 22 (0.75) | 0.35 (0.19,0.64) ** | 11 (0.41) | 0.35 (0.22,0.56) ** | 29 (0.66) | 0.73 (0.19,2.80) | 4 (0.33) |
| √ | √ |  | √ | 0.47(0.16,1.35) | 368 (6.56) | 0.42 (0.11,1.63) | 187 (6.37) | 0.56 (0.10,3.09) | 181 (6.76) | 0.40 (0.13,1.21) | 255 (5.79) | / | 113 (9.39) |
| √ |  | √ | √ | 0.31(0.18,0.56) ** | 215 (3.83) | 0.36 (0.13,0.96) * | 81 (2.76) | 0.26 (0.12,0.59) ** | 134 (5.01) | 0.35 (0.20,0.61) ** | 171 (3.88) | 0.32 (0.06,1.68) | 44 (3.65) |
| √ | √ | √ | √ | 0.32(0.21,0.50) ** | 497 (8.86) | 0.36 (0.20,0.66) ** | 171 (5.83) | 0.28 (0.14,0.57) ** | 326 (12.18) | 0.30 (0.19,0.48) ** | 395 (8.96) | 0.81 (0.17,3.93) | 102 (8.47) |

Odds ratio and 95% CI were calculated. P-values less than 0.05 (p < 0.05) were considered significant. * Represented p < 0.05, ** Represented p < 0.01. N: number, CI: confidence interval.
