# Supplementary material for: Association between healthy lifestyle combinations and periodontitis in NHANES
Source: BMC Oral Health. 2024 Feb 4;24:182. doi: 10.1186/s12903-024-03937-z (PMC10840229; doi:10.1186/s12903-024-03937-z)
Supplement: Supplementary file 3 — Table S3: Linear regression model between healthy lifestyle factors and clinical attachment loss of the heaviest site in periodontitis group. No covariates were adjusted in Model 1. Model 2 was adjusted for age and gender. Model 3 was adjusted for ethnicity, family income-to-poverty ratio, educational level, and history of diabetes on the base of Model 2. P-values less than 0.05 (p < 0.05) were considered significant. CAL: clinical attachment loss; CI: confidence interval [file 12903_2024_3937_MOESM3_ESM.docx]

**Table S2** Linear regression model between healthy lifestyle factors and clinical attachment loss of the heaviest site in periodontitis group.

|  |  | **The CAL of the heaviest site** | | |
| --- | --- | --- | --- | --- |
|  |  | **β** | **95%CI** | **P-value** |
| **Healthy lifestyle factors** | Model 1 | -0.19 | (-0.23, -0.15) | <0.01 |
|  | Model 2 | -0.20 | (-0.25, -0.16) | <0.01 |
|  | Model 3 | -0.11 | (-0.15, -0.06) | <0.01 |

No covariates were adjusted in Model 1. Model 2 was adjusted for age and gender. Model 3 was adjusted for ethnicity, family income-to-poverty ratio, educational level, and history of diabetes on the base of Model 2. P-values less than 0.05 (*p* < 0.05) were considered significant. CAL: clinical attachment loss; CI: confidence interval.
