# Supplementary figures and images for: Association between healthy lifestyle combinations and periodontitis in NHANES
Source: BMC Oral Health. 2024 Feb 4;24:182. doi: 10.1186/s12903-024-03937-z (PMC10840229; doi:10.1186/s12903-024-03937-z)

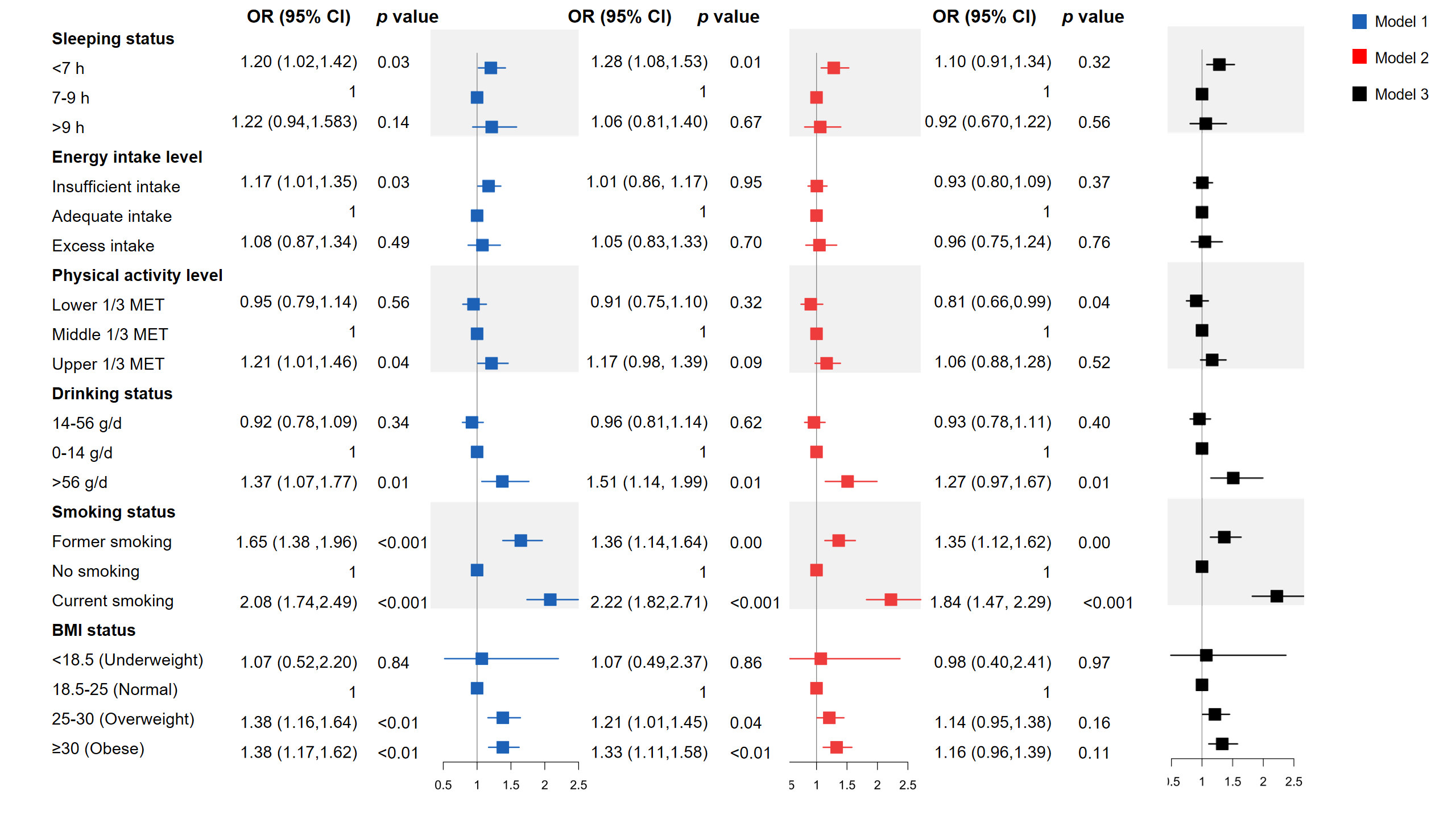

Supplement: Supplementary file 4 — Figure S1: Logistic regression models for associating single lifestyle factor with periodontitis prevalence. No covariates were adjusted in Model 1(Black). Model 2 (Blue) was adjusted for age and gender. Model 3 (Red) was adjusted for ethnicity, family income-to-poverty ratio, educational level, and history of diabetes on the base of Model 2. Odds ratio and 95% CI were calculated. P-values less than 0.05 (p < 0.05) were considered significant. CI: confidence interval. [file 12903_2024_3937_MOESM4_ESM.tif]
